# Real-time point of care microcirculatory assessment of shock: design, rationale and application of the point of care microcirculation (POEM) tool

**DOI:** 10.1186/s13054-016-1492-1

**Published:** 2016-09-30

**Authors:** David N. Naumann, Clare Mellis, Shamus L. G. Husheer, Philip Hopkins, Jon Bishop, Mark J. Midwinter, Sam D. Hutchings

**Affiliations:** 1 NIHR Surgical Reconstruction and Microbiology Research Centre, Queen Elizabeth Hospital, Birmingham, B152TH UK; 2 Kings College Hospital, Denmark Hill, London, UK; 3 Heartfelt Technologies Ltd, Fen House Fen Road, Cambridge, UK

**Keywords:** Microcirculation, Shock, Circulation, Sepsis, Hemorrhage, Point-of-care

## Abstract

**Background:**

Despite over a decade of research and technological advances, sublingual microcirculatory monitoring has not yet reached clinical utility. Offline analysis is time consuming and occurs away from the patient. A system to assess the microcirculation at the point of care is desirable. We present a novel 5-point grading system (the point of care microcirculation (POEM) scoring system) that can be used at the point of care during non-invasive sublingual microcirculatory monitoring.

**Methods:**

The POEM score is an ordinal scale from 1 (worst) to 5 (best), based on a composite assessment of flow and heterogeneity of four individual sublingual video-microscopy clips. Thirty-two healthcare professionals were trained in how to assign POEM scores. Following training they assigned scores to five test sequences (each consisting of four video clips). They were blinded to clinical status. Inter-user consistency and agreement were assessed using intra-class correlation coefficient (ICC) analysis. In addition, blinded expert scores for 68 video clips were compared to offline computer analysis using traditional microcirculatory parameters including total vessel density (TVD), perfused vessel density (PVD), proportion of perfused vessels (PPV), microcirculatory flow index (MFI) and microcirculatory heterogeneity index (MHI). The time taken to assign each was recorded.

**Results:**

Participants showed good inter-rater consistency (ICC 0.83, 95 % CI 0.626, 0.976) and agreement (ICC 0.815, 95 % CI 0.602, 0.974) for assigned POEM scores. Expert scoring of videos correlated with offline values for PVD (*R*^*2*^ = 0.39; *p* < 0.05), PPV (*R*^*2*^ = 0.71; *p* < 0.001), MFI (*R*^*2*^ = 0.75; *p* < 0.001), and MHI (*R*^*2*^ = 0.68; *p* < 0.001). POEM scores took less time to assign than conventional offline computer analysis (2 minutes versus 44 minutes).

**Conclusion:**

We present for the first time a novel 5-point ordinal scale of microcirculatory flow and heterogeneity that can be used at the point of care. It has minimal inter-user variability amongst healthcare professionals after just 1 hour of training. POEM scores take a short time to assign, and correspond well to traditional offline computer-analyzed parameters.

**Electronic supplementary material:**

The online version of this article (doi:10.1186/s13054-016-1492-1) contains supplementary material, which is available to authorized users.

## Background

As the microcirculation is the anatomical location of oxygen and substrate exchange, its behavior during shock is of interest to those involved in patient resuscitation. The term “hemodynamic coherence” has been used to describe a situation in which resuscitation aimed at restoring systemic hemodynamic parameters (such as cardiac output) also makes a corresponding improvement to the microcirculation [1]. Some pathological circumstances such as sepsis may cause an imbalance between global and microcirculatory parameters so that the microcirculation no longer corresponds to the macrocirculation (i.e., loss of hemodynamic coherence); in this circumstance goal-directed resuscitation targeted towards global parameters may lead to harm [1]. There is some evidence that when microcirculatory flow is impaired during circulatory shock it may not be restored even when blood pressure is improved [2]. Microcirculatory parameters may also predict clinical outcomes better than global measurements in sepsis [3] and traumatic hemorrhagic shock [4]. Improvement of microcirculatory parameters during resuscitation may also predict better outcomes following major surgery [5] and sepsis [6].

The use of hand held non-invasive sublingual video-microscopes (such as sidestream dark field (SDF) or incident dark field (IDF) microscopy) has allowed researchers to study microcirculatory flow in vivo, both in experimental models and in patients. However, despite over a decade of detailed investigations and demonstrations of the rationale of monitoring the microcirculation, this technology and associated techniques have still not advanced from the research to clinical domains. A major limitation in the use of current video-microscope technology is that analysis depends on the capture of video clips that require offline analysis. This takes a considerable amount of time (after the clinical window of diagnostic utility). Real-time point of care automated (computerized) analysis has not yet been validated against traditional offline analysis. Even if computerized automated analysis were to yield accurate, validated parameters, the clinical applicability is unlikely to be meaningful without user interpretation and some form of clinical grading system that might determine particular therapeutic pathways based on target readings. This is because such a system may yield parameters of unknown clinical relevance. What is required in real-life clinical practice is a simple grading system that acts as a trigger or guide for the delivery of particular interventions.

Here we describe for the first time a 5-point ordinal grading scale of microcirculatory function based on a composite of flow and heterogeneity in vessel segments viewed by sublingual video-microscopy. The score is relatively simple and can be assigned at the point of care. It may be one way of facilitating goal-directed therapy using microcirculatory parameters.

## Methods

### Design of the point of care microcirculation grading system

**Fig. 1:**
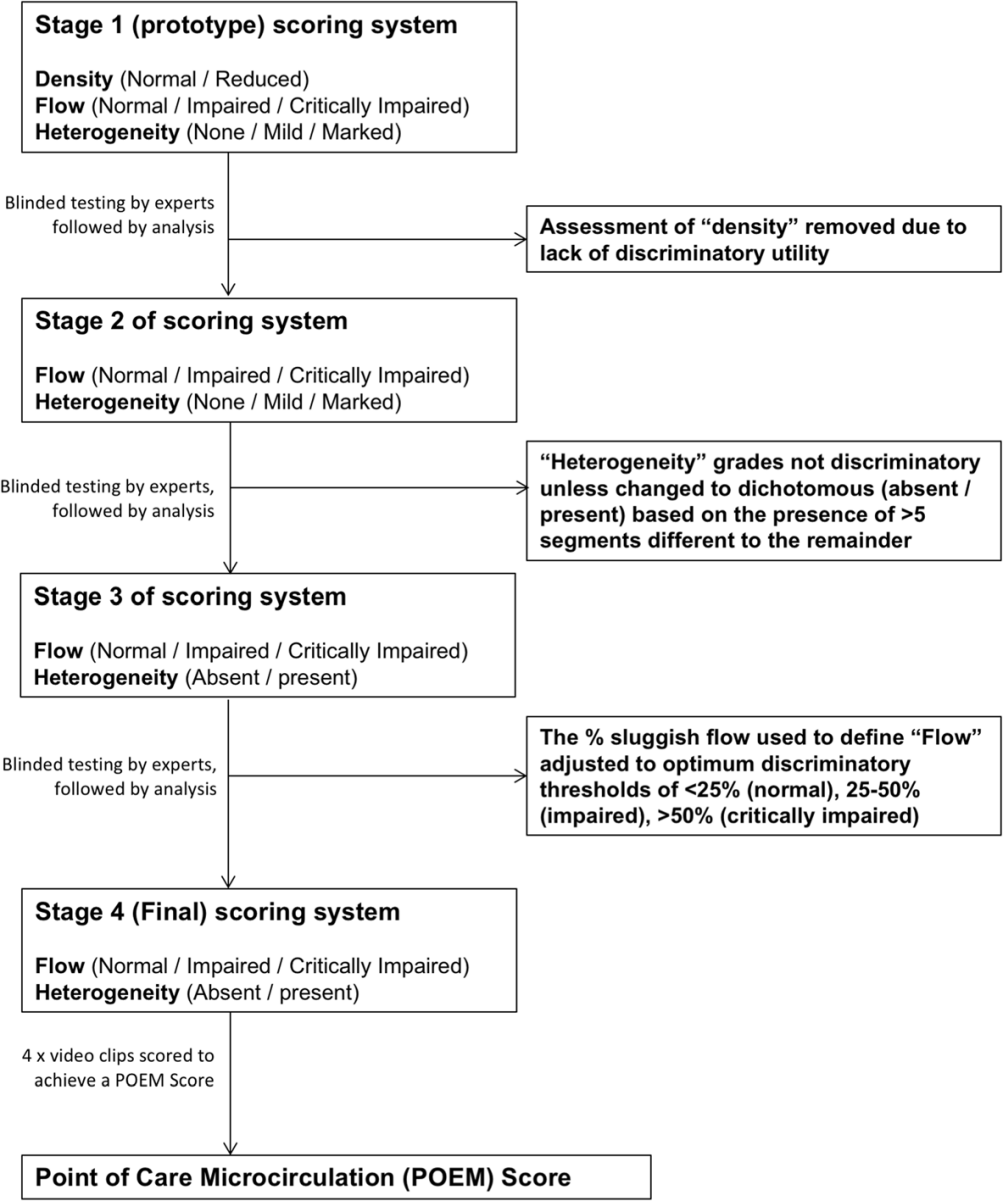
Schematic flow diagram of the stages in development of the final point of care microcirculation (*POEM*) scoring system

A schematic diagram (Fig. 1) summarizes the stages in the design of the point of care microcirculation (POEM) score. The scoring system is based on the premise that flow and heterogeneity of vessel segments are the key components of interest. The final POEM score does not account for vessel density, because early trials of the scoring system that incorporated assessment of density did not demonstrate its discriminatory utility.

### Assigning a POEM score

The final POEM scoring system is a 5-point ordinal scale that integrates assessments of both flow and heterogeneity (Table 1). It enables a user to assess real-time microcirculatory videos obtained during sublingual IDF microscopy. It is derived from the assessment of four video clips from the same patient at the same time point (as a recommendation of three to five video clips corresponds to the consensus opinion in traditional analysis [7]). For each of the four individual video clips, the user determines the flow and heterogeneity as such.

**Table 1:** Point of care microcirculation (POEM) grade and corresponding definitions

POEM score	Microcirculatory function
5	Normal flow^a^, with no heterogeneity^d^
4	Normal flow^a^, with mild heterogeneity^e^
3	Normal flow^a^, with marked heterogeneity^f^
2	Impaired flow^b^ (heterogeneity is also present)
1	Critically impaired flow^c^ (heterogeneity is also present)

^a^Less than 25 % of vessel segments in view are sluggish/stopped; ^b^25–50 % of vessel segments in view are sluggish/stopped; ^c^more than 50 % of vessel segments in view are sluggish/stopped;

^d^0 or 1 clips have heterogeneity present; ^e^2 or 3 clips have heterogeneity present; ^f^all 4 clips have heterogeneity present

- Flow
  1. Normal: <25 % of vessel segments in view are sluggish/stopped
  2. Impaired: 25–50 % of vessel segments in view are sluggish/stopped
  3. Critically impaired: >50 % of vessel segments in view are sluggish/stopped
- Heterogeneity
  Only if the user determines that a clip has “normal” overall flow are they prompted to also assign whether heterogeneity is present or absent. This is because during the pilot phase of developing the scoring system (Fig. 1) we found that heterogeneity was universally present in clips with “impaired” and “critically impaired” overall flow (i.e., having an assessment of heterogeneity for such scenarios did not make any difference overall). Heterogeneity is determined for “normal” clips as “present” if >5 vessel segments demonstrate different flow to the remainder. The threshold for 5 vessels segments was used because this was found to be the most discriminating (Fig. 1).

Each individual video clip is therefore assigned either: “critically impaired”, “impaired”, “normal flow with heterogeneity”, or “normal flow without heterogeneity”.

### POEM score calculation

**Fig. 2:**
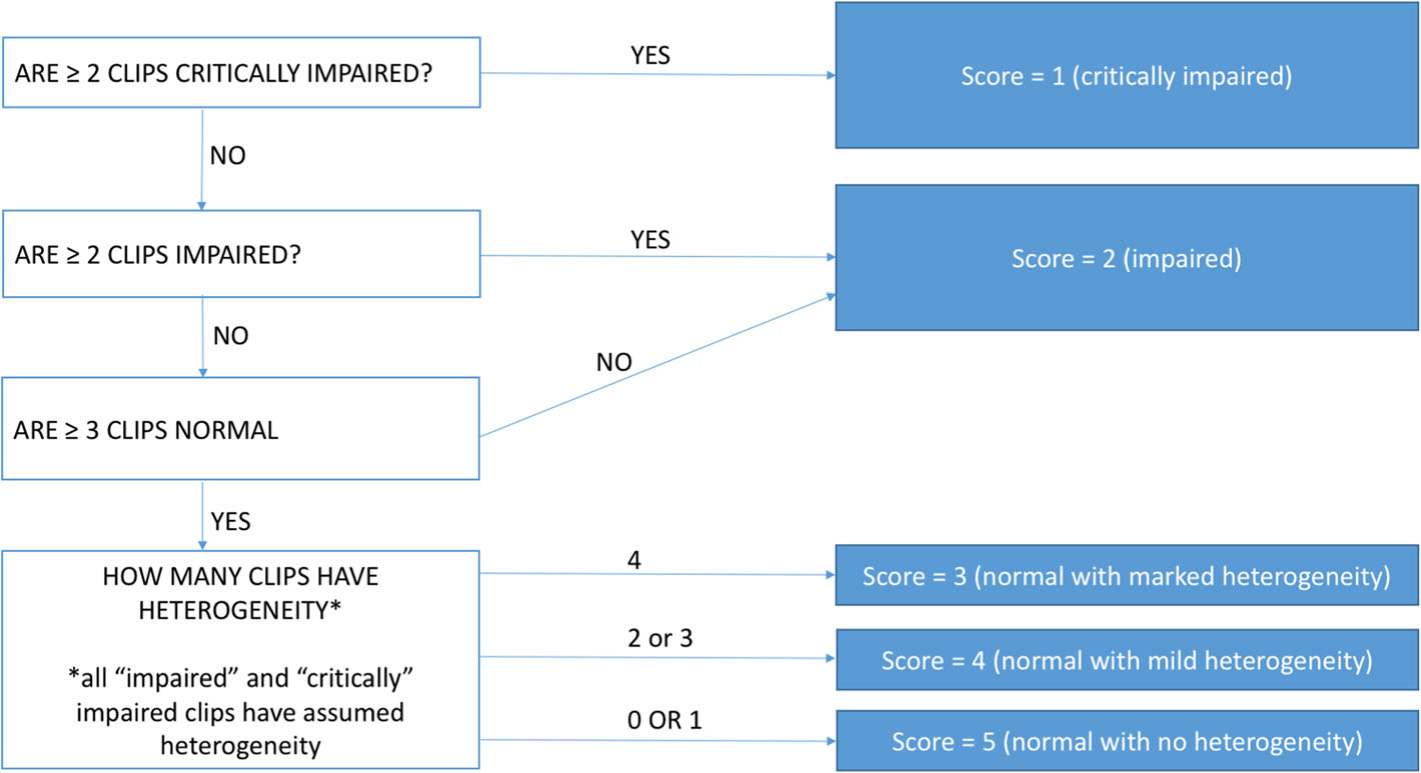
Algorithm for overall point of care microcirculation (POEM) score using the parameters from four video clips

It is the combination of all four video clips that gives the overall POEM score as per the algorithm in Fig. 2. In short, if two or more clips have “critically impaired” flow, then the overall POEM score is 1 (critically impaired). If two or more clips have “impaired” flow then the overall POEM score is 2 (impaired). If three or more clips have “normal” flow then they can be one of three different scores: POEM score 3 (normal with marked heterogeneity) if all four clips show heterogeneity; POEM score 4 if two or three clips show heterogeneity; and POEM Score 5 if one or fewer clips have heterogeneity.

### Online tool

**Fig. 3:**
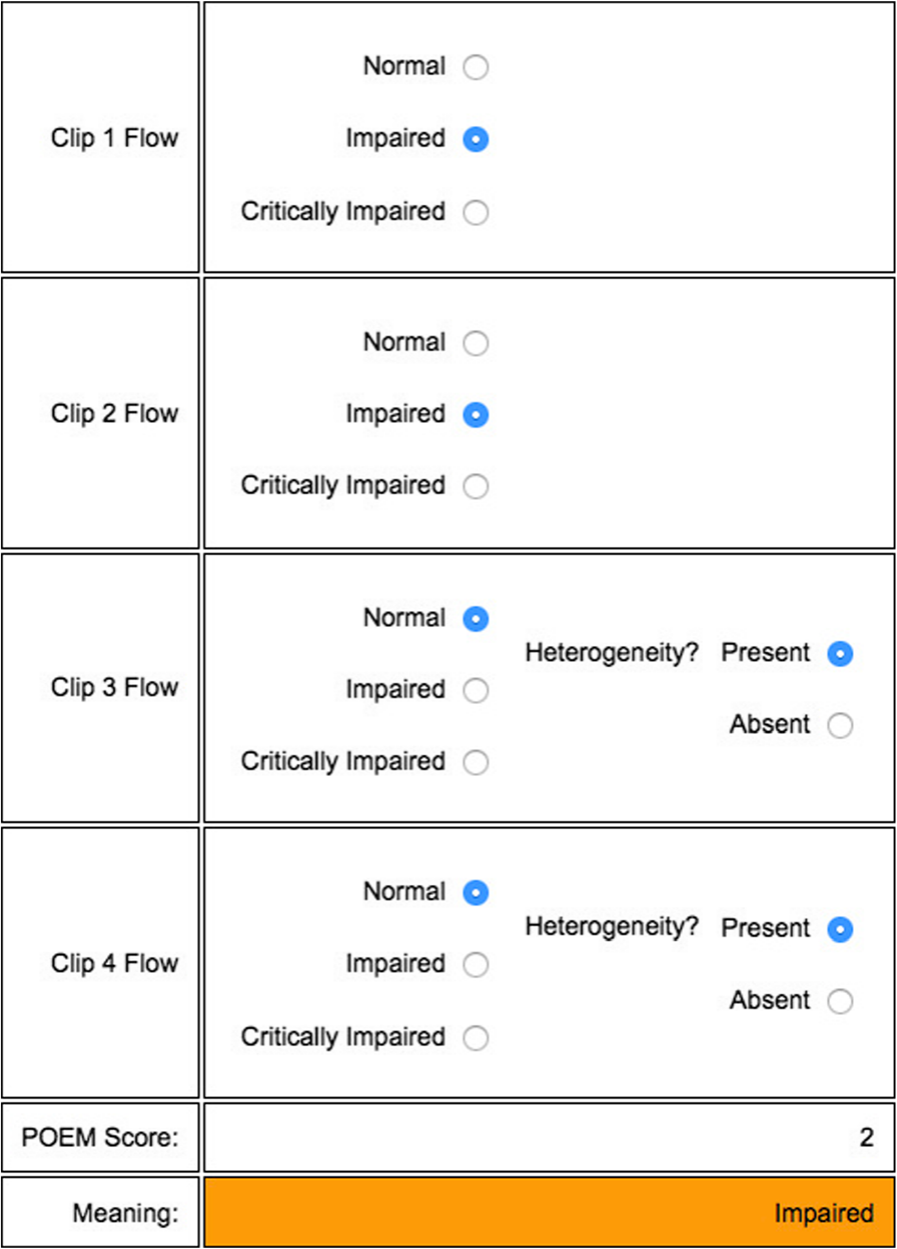
An example of a point of care microcirculation (*POEM*) grade being assigned using the online tool. In this case the overall POEM score is 2, indicating “impaired” flow

An online tool (http://www.POEMscore.com) can be used to perform the calculation for the POEM score based on the scores for all four video clips with minimal effort. An example of an online POEM score calculation is shown in Fig. 3, and is based on the algorithm illustrated in Fig. 2.

### Validation of the scoring system by healthcare professionals

A group of 32 healthcare professionals volunteered as study participants to assess the utility of the POEM score. These participants were from two UK collaborating sites (University Hospitals Birmingham NHS Foundation Trust, Birmingham, UK, and King’s College Hospital NHS Foundation Trust, London, UK). All participants were current intensive care clinicians or nurses and had not previously used sublingual microcirculatory monitoring or interpreted video-microscopy clips.

#### Training

All study participants undertook a standardized 60-minute interactive training session using 18 slides and a pre-selected set of videos of varying microcirculatory dysfunction as examples (Additional file 1). All video clips were obtained from the Microshock study [8], from patients with traumatic hemorrhagic shock (mixed blunt and penetrating trauma) and taken on day 0 or day 1 of their hospital stay. The aim of the training session was to teach the participants how to assign “normal”, impaired”, or “critically impaired” flow to individual video clips, and to determine whether heterogeneity was “present” or “absent” for “normal” clips. Teaching sessions were delivered by subject matter experts (DNN and SDH). The participants were given an opportunity to ask questions and look again at some example videos before being asked to score the test sequence of video clips.

#### Testing

Straight after the training session, the participants were asked to view and score 5 video sequences (corresponding to five different patients), each of which consisted of 4 video clips (20 clips in total). These videos were taken from the Microshock study [8], and had been recorded using an IDF video microscope recently validated for use in shock states [9], and all had been assessed as high quality according to guidelines [10]. The participants were blinded to each others’ scores and the clinical status of the patients. They were allowed up to 2 minutes for each individual video clip, which was played in “loop” until the allocated time was reached. Once all 4 clips had been watched and scored for a given sequence, the participants were not allowed to revise their scores.

### Human versus offline computer analysis of video clips

A random selection of 68 individual video clips, acquired by sublingual IDF video-microscopy during the Microshock study from eight patients at 15 time points [8] were analyzed offline using Automated Vascular Analysis V.3.02 (Microvision Medical, The Netherlands). Semi-automated analysis was utilized as described in greater detail elsewhere [8] (fully automated analysis was not performed). All video clips were rated for quality [10], and then semi-quantitative data were recorded for each video clip according to consensus guidelines [7]. These included total vessel density (TVD), perfused vessel density (PVD), proportion of perfused vessels (PPV), and microcirculatory flow index (MFI) for individual clips; and microcirculatory heterogeneity index (MHI) for each time point. Each individual video clip was assigned a random number at time of analysis, and played in random order for an expert user (SDH) to grade according to the POEM scoring tool. Therefore the expert user was blinded to both the computer analysis parameters and the clinical status of the patients.

Using the assessments from these individual video clips, POEM scores were applied to all 15 time points according to the algorithm in Fig. 2. No time limit was imposed for the expert to assign POEM scores or computer analysis. The time taken to assign POEM scores and perform computer analysis was recorded.

### Data analysis

Inter-user variability for the ordinal 5-point scale was assessed using the intra-class correlation coefficient (ICC) in terms of both consistency (e.g. do participants tend to score item A higher than item B but lower than item C) and agreement (e.g. do participants all tend to give a score of X to item A and a score of Y to item B). Values are presented with 95 % confidence intervals (CI). Analysis assumed a two-way model (where both the video clips and individual participants are regarded as random samples from a potential larger pool of video clips and participants). Naïve participant scores were also compared to expert scores by subtracting the true expert score from the observed rater scores and then perform a linear regression on these “error scores”, including rater and video sequence as independent predictors.

Values from computer analysis (PVD, TVD, PPV, MFI, and MHI) were compared to ordinal POEM scores using linear regression. Further comparison was made between computer analysis parameters and both individual video scores and POEM scores as categorical variables (using Kruskal-Wallis analysis followed by stepwise paired analysis using Dunn’s multiple comparisons test). A *p* value <0.05 was considered significant.

## Results

### Study participants

There were 32 study participants, including 12 consultants, 18 training-grade doctors, and 2 intensive care nurses. All of these participants worked in their hospital ICUs. None had used sublingual video-microscopy before, and all were naïve to IDF video analysis. All participants completed the training and assessment sessions. The expert user (SDH) who conducted the offline computer analysis and POEM scoring has analyzed over 1000 video-microscopy clips.

### Inter-user variability

When the naïve user POEM scores were analyzed to determine the inter-user variability between clips and between each other, the ICC values for consistency and agreement were 0.83 (95 % CI 0.626, 0.976) and 0.815 (95 % CI 0.602, 0.974), respectively. From the analysis of variance between users and expert, there was greater agreement for “critically impaired” and “normal” but higher variability for the scores in between; both item and rater were significant predictors of the level of error in the scores when compared to expert (*p* < 0.001 and *p* = 0.046, respectively).

### Human versus computer analysis of video clips

**Fig. 4:**
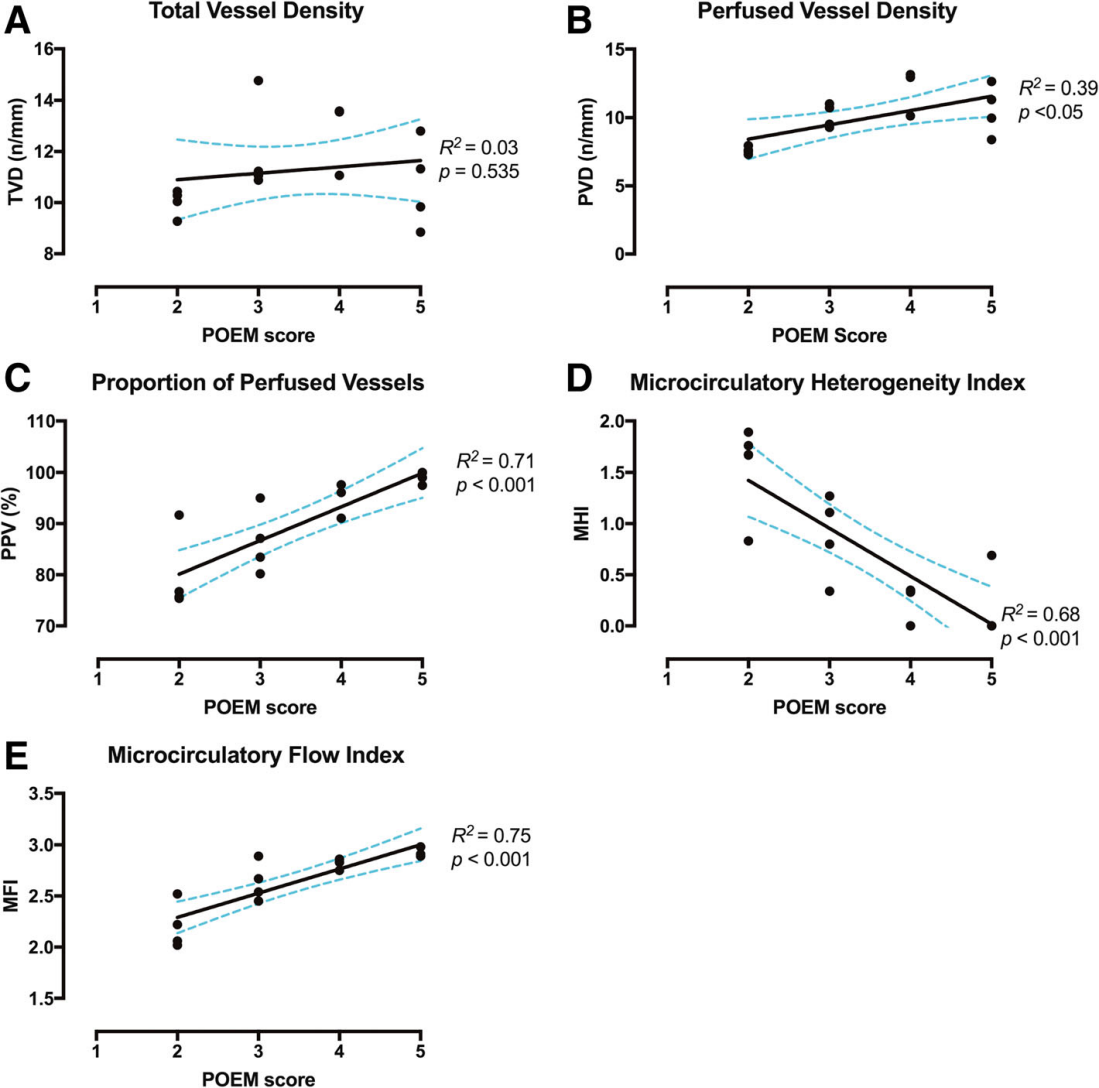
Relationship between traditional offline computer analysis and individual point of care microcirculation (*POEM*) scores. *Dashed lines* indicate 95 % confidence interval. **a** *TVD* total vessel density, **b** *PVD* perfused vessel density, **c** *PPV* proportion of perfused vessels, **d** *MHI* microcirculatory heterogeneity index, **e** *MFI* microcirculatory flow index

When traditional offline computer analysis parameters were compared to expert assigned POEM scores there was good correlation with PPV (*R*^*2*^ = 0.71; *p* < 0.001), PVD (*R*^*2*^ = 0.39; *p* < 0.05), MFI (*R*^*2*^ = 0.75; *p* < 0.001), and MHI (*R*^*2*^ = 0.68; *p* < 0.001), but not TVD (*R*^*2*^ = 0.03; *p* = 535) (Fig. 4).

In addition, when offline computer analysis parameters were compared to expert assigned grades for the 68 individual video clips, these scores corresponded well to perfusion (PPV and PVD) and flow (MFI) parameters, but there was no statistically significant relationship to the pure density parameter (TVD) (Additional file 2: Figure S1). When computer analysis of heterogeneity (MHI) and flow (MFI) were compared to POEM scores for the 15 time points, there was also a significant association (Additional file 3: Figure S2). When the timings were compared between the assignment of POEM scores and completion of computer analysis they were 2 minutes and 44 minutes, respectively (*p* < 0.001).

## Discussion

The current study reports the first use of a novel 5-point grading system (the POEM score) for the hybrid assessment of microcirculatory flow and heterogeneity. Naïve users can be trained to assess the microcirculation using this system, and have produced scores with relative consistency and agreement even after just one teaching session. Furthermore the scoring is in keeping with offline computer-generated analysis of flow, perfusion and heterogeneity and takes several minutes rather than an hour to perform. We present a user-friendly, straightforward tool that may be used as a point of care test in the observation of patients who are being treated for shock. Clinical validation and further examination of the real-time applicability are required before the 5-point grading system can be introduced into clinical practice, in particular in relation to other parameters such as CO_2_ gap and lactate. The POEM scoring system has the potential to alert the clinician to lack of hemodynamic coherence, and also to the degree of microcirculatory disruption in terms of flow and heterogeneity.

The semi-quantitative “Boerma Method” of assigning MFI values during sublingual video-microscopy [11] has the potential to be used in real-time. Although it is usually performed offline as part of the full panel of agreed parameters [7], it has also been used at the bedside as a real-time measurement of microcirculatory flow with good reliability when compared to offline computer analysis [12, 13]. The current study shows that POEM scores correspond well to MFI scores from the same video sequences for both individual videos and patient time-points. The potential advantage of the POEM scores over MFI is that it also appears to correspond well to traditional values of heterogeneity (MHI), flow (PPV) and PVD (composite flow and density) rather than flow alone. The MFI approach involves superimposing a quadrant grid over the video images and then grading the MFI score for each quadrant before performing a calculation to determine the difference in minimum and maximum MFI scores compared to the average. By contrast the POEM score requires the user to identify whether more than five vessel segments in the entire field have flow abnormalities, removing the potentially artificial quadrant approach. We consider that such a technique is easier to perform for naive users, and has the advantage of producing a single composite ordinal score that still gives accurate information about the state of overall flow and flow heterogeneity.

Although heterogeneity is usually presented as MHI by calculating the differences in MFI values, the POEM score uses a simplified method that does not require calculation, in order to make it easier to use. The clinician is only required to determine whether there are five vessel segments different to the rest in the field of view, regardless of quadrant. As POEM scores reflect MHI well, this method may not necessarily be objectively precise but appears to be suitable in determining the level of heterogeneity for the purposes of a 5-point ordinal scale as well as full traditional MHI calculations.

Recently some investigators have demonstrated that visual inspection by clinicians may have good agreement with more detailed offline computer analysis, making the prospect of point of care microcirculatory analysis more of a realistic prospect [14]. A further study has demonstrated feasibility of bedside microcirculatory monitoring by critical care nursing staff [15]. If a clinician is to utilize sublingual microcirculatory monitoring to direct treatment in real time, the video microscopy must be interpreted in a validated, graded, replicable manner with minimum inter-user variability. If the clinician can determine that the patient has lost hemodynamic coherence using a grading system of microcirculatory dysfunction, then alterations to therapy might be made that otherwise would have been guided by macrocirculatory parameters alone. A grading system of severity of microcirculatory dysfunction may aid in diagnosis, prognosis and management.

Although it has been an aspiration for some time, the real-time monitoring of the microcirculation at the point of care has been more of a futuristic prospect [16, 17] rather than a present-day clinical tool in the armory of the clinician. It is timely to bring the available expertise and technology into clinical practice. Technology has advanced so that handheld, ergonomic instruments (such as the IDF videomicroscope (Cytocam, Braedius Medical B.V., Huizen, The Netherlands)) can be used to visualize the sublingual microcirculation with ease and speed. There has been a decade of research since the 2006 microcirculation consensus meeting [7], and yet microcirculatory monitoring has been confined to the realm of research during that time. The microcirculation community eagerly awaits automated computer-generated analysis so that the microcirculation can be assessed without the requirement for offline analysis. However, it is likely even with such technology that a more simplified clinical grading system will be of maximal utility for the clinician at the point of care. Essentially the clinician’s two primary aims when monitoring the microcirculation are (i) to determine whether there is loss of hemodynamic coherence; and (ii) to determine the degree of microcirculatory disruption. These factors have the potential to guide therapy.

Traditional offline measurements of microcirculatory function will be of continued value in the research context for studies that utilize microcirculatory parameters as resuscitation outcome measures. They will also be important when testing particular therapeutic measures following shock, and in providing diagnostic information (such as type of shock and classification of subtypes), and in tailoring therapy to specific requirements. The benefit of the POEM study in the clinical context is that it is faster to obtain, can more easily be used in real time, and has the potential to guide and direct therapy at the bedside. Rather than replacing traditional numerical values, the POEM score is designed so that it can be used concurrently. One of the potential uses of the POEM score is as an early warning marker for microcirculatory dysfunction and loss of coherence. More detailed assessment of the microcirculation will still be required to determine the precise nature of microcirculatory dysfunction.

### Future avenues of research

The POEM score does not replace traditional parameters in their role as research endpoints. Rather, it brings the overall assessment of the microcirculation forward to the point of care, in order to act as guidance of initial therapy. It is not currently known whether this will improve outcomes for patients, but future prospective randomized studies may wish to test the utilization of the POEM score versus standard practice in the management of patients in shock (such as septic or traumatic hemorrhagic shock). Furthermore, it is possible that the clinician might direct resuscitative fluids in a manner that addresses microcirculatory dysfunction when such a phenomenon is detected at the point of care (rather than being falsely reassured by satisfactory global hemodynamic parameters). Recent studies have reported that the delivery of plasma appears to have a restorative effect on the endothelial glycocalyx and ameliorate the endotheliopathy of trauma [18, 19]. Could the detection of microcirculatory failure and subsequent delivery of plasma in a circumstance where such therapy would be omitted be beneficial to patients? Such a question remains unanswered in a clinical context and may be a promising avenue for future research. However such research depends entirely on a rapid and reproducible assessment of the microcirculation at the point of care - something that the POEM assessment tool may provide.

The introduction of user-dependency on a point of care test is not without precedence; cardiac echo and ultrasonography are some obvious examples of techniques that require high-quality assessment by expert users for reliable readings. Trained users are accustomed to the practice of a fast, efficient assessment of patients in the Emergency Department (ED) in the case of focused assessment with sonography in trauma (FAST). Quality assurance and regular training, experience and revalidation should be of upmost importance amongst microcirculation monitoring users if their point of care assessment were to enter the domain of clinical utility. The current study did not test the level of skill fade or utility of the scoring system after a time interval, but these are aspects of training and professional development that would require attention if the POEM score were to be used clinically. Similarly, devices and technology would need to come under the scrutiny of clinical point of care governance to ensure safety and consistency in clinical practice.

If real-time microcirculatory assessment is to be performed at the bedside, then quality assessment of video clips must also be undertaken before an assessment of flow and heterogeneity can be made. The current study only utilized videos that were high quality; whether poorer quality videos in a real-world scenario might lead to more variability in grading is unknown. The assessment of quality needs to be consistent, and reproducible, and with little inter-user variability. In particular attention should be paid to guarding against pressure artifact, perhaps by ensuring that there are visible flowing venules within the field of view.

A decision was made early in the formation of the POEM score to not incorporate an assessment of vessel density, instead favoring assessments of flow and heterogeneity. Assessment of density was not a good discriminator between video clips when testing the early forms of the scoring system. Early pilot testing of the scoring system also showed that density assessment was difficult to apply in a manner that would accurately reflect TVD values. Nevertheless, POEM scores do correspond well to PVD, which is in itself a mixed density and perfusion variable. As the POEM score corresponds well to PPV, PVD, MFI, and MHI, it is the first tool to yield a composite assessment of flow and heterogeneity and also yield potentially meaningful data on perfusion and perfused vessel density.

### Limitations

None of the POEM study subjects were physically next to a patient’s bedside when assigning POEM scores. It is unknown whether this physical proximity and patient contact would influence the scoring or performance of the test. Instead, the study subjects were blinded to patient status in order to establish the scoring system without any bias that clinical exposure might bring. Further validation at the patient’s bedside may be warranted in future studies that utilize the POEM score.

This study utilized video-microscopy clips of patients with traumatic hemorrhagic shock. Further validation in other clinical scenarios such as sepsis and following major surgery are required in order to determine whether this scoring tool has more generalizable utility.

Although there is a good association between POEM assessments and PVD, the POEM scoring system does not take into account pure density parameters of the microcirculation (such as TVD). This may limit its usefulness in determining the etiology of the microcirculatory derangement. Measures to determine density parameters must rely on alternative techniques, which are currently not available at the point of care.

## Conclusions

A new 5-point ordinal scoring system of microcirculatory flow and heterogeneity has been tested amongst healthcare professionals at two large UK teaching hospitals, and has relatively high consistency and agreement even after just 1 hour of training. POEM scores take a matter of minutes to assign, and correspond well to computer-analysis variables of flow, perfusion and heterogeneity. We present for the first time a point of care microcirculatory assessment tool that is quick, reliable, and gives potentially meaningful clinical parameters that might guide resuscitation. Prospective randomized trials utilizing goal directed therapy targeted at the POEM score are required to test its real-life clinical utility.

## Additional files

Additional file 1: POEM training presentation. (PPTX 464396 kb) Additional file 2: Figure S1. Relationship between traditional offline computer analysis and individual scores for video clips using the POEM score as a categorical variable (**p* < 0.05; ***p* < 0.01; ****p* < 0.001). (TIFF 744 kb) Additional file 3: Figure S2. Relationship between offline computer analysis of microcirculatory heterogeneity and flow indexes and POEM scores with the POEM score as a categorical variable (**p* < 0.05; ***p* < 0.01). (TIFF 278 kb)

## Abbreviations

ED: Emergency Department
FAST: Focused assessment with sonography in trauma
ICC: Intra-class correlation coefficient
ICU: Intensive Care Unit
IDF: Incident dark field
MFI: Microcirculatory flow index
MHI: Microcirculatory heterogeneity index
POEM: Point of care microcirculation
PPV: Proportion of perfused vessels
PVD: Perfused vessel density
SDF: Sidestream dark field
TVD: Total vessel density
