# Supplementary figures and images for: Real-time point of care microcirculatory assessment of shock: design, rationale and application of the point of care microcirculation (POEM) tool
Source: Crit Care. 2016 Sep 30;20:310. doi: 10.1186/s13054-016-1492-1 (PMC5045597; doi:10.1186/s13054-016-1492-1)

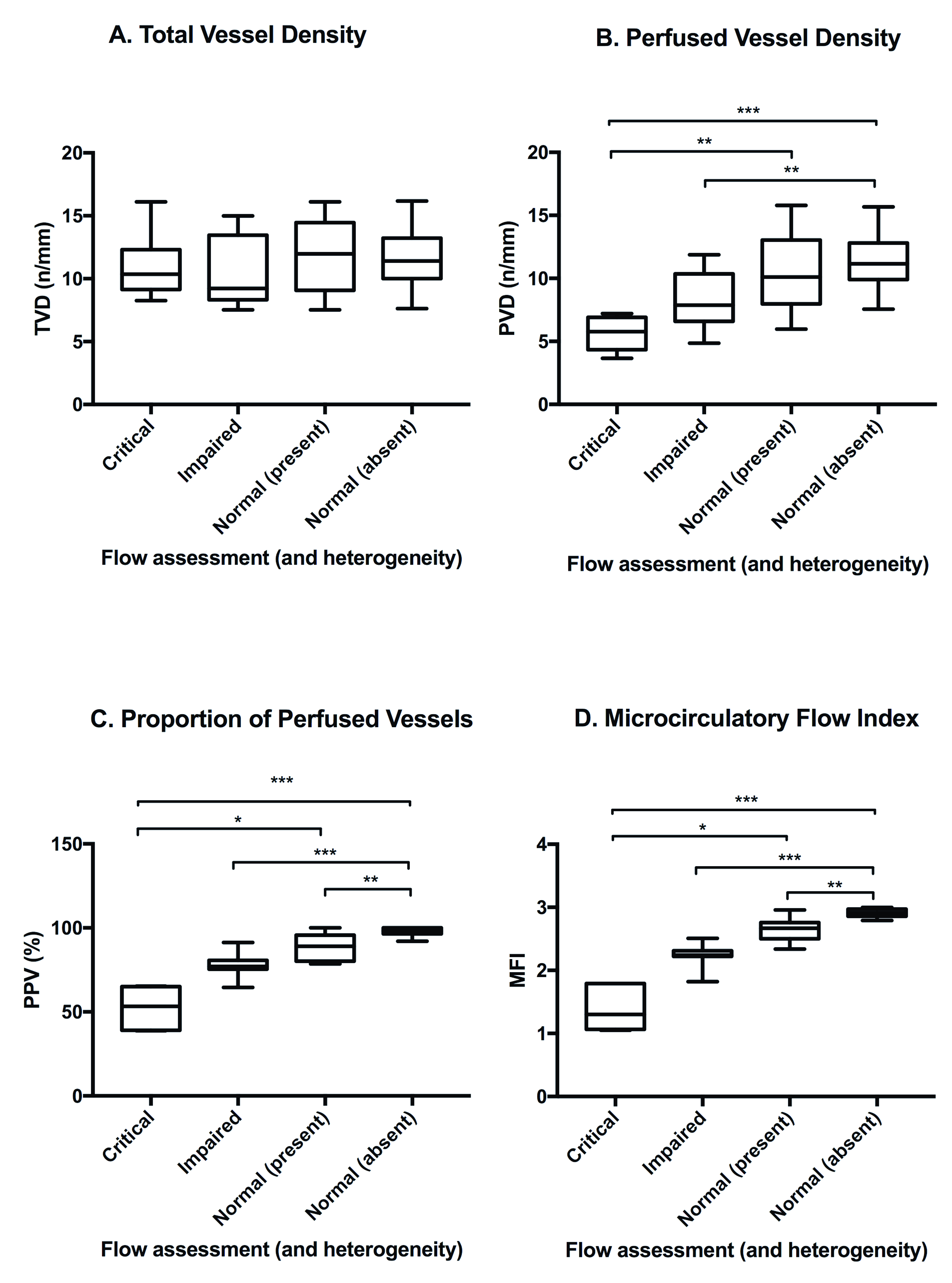

Supplement: Additional file 2: Figure S1. — Relationship between traditional offline computer analysis and individual scores for video clips using the POEM score as a categorical variable (*p < 0.05; **p < 0.01; ***p < 0.001). (TIFF 744 kb) [file 13054_2016_1492_MOESM2_ESM.tiff]

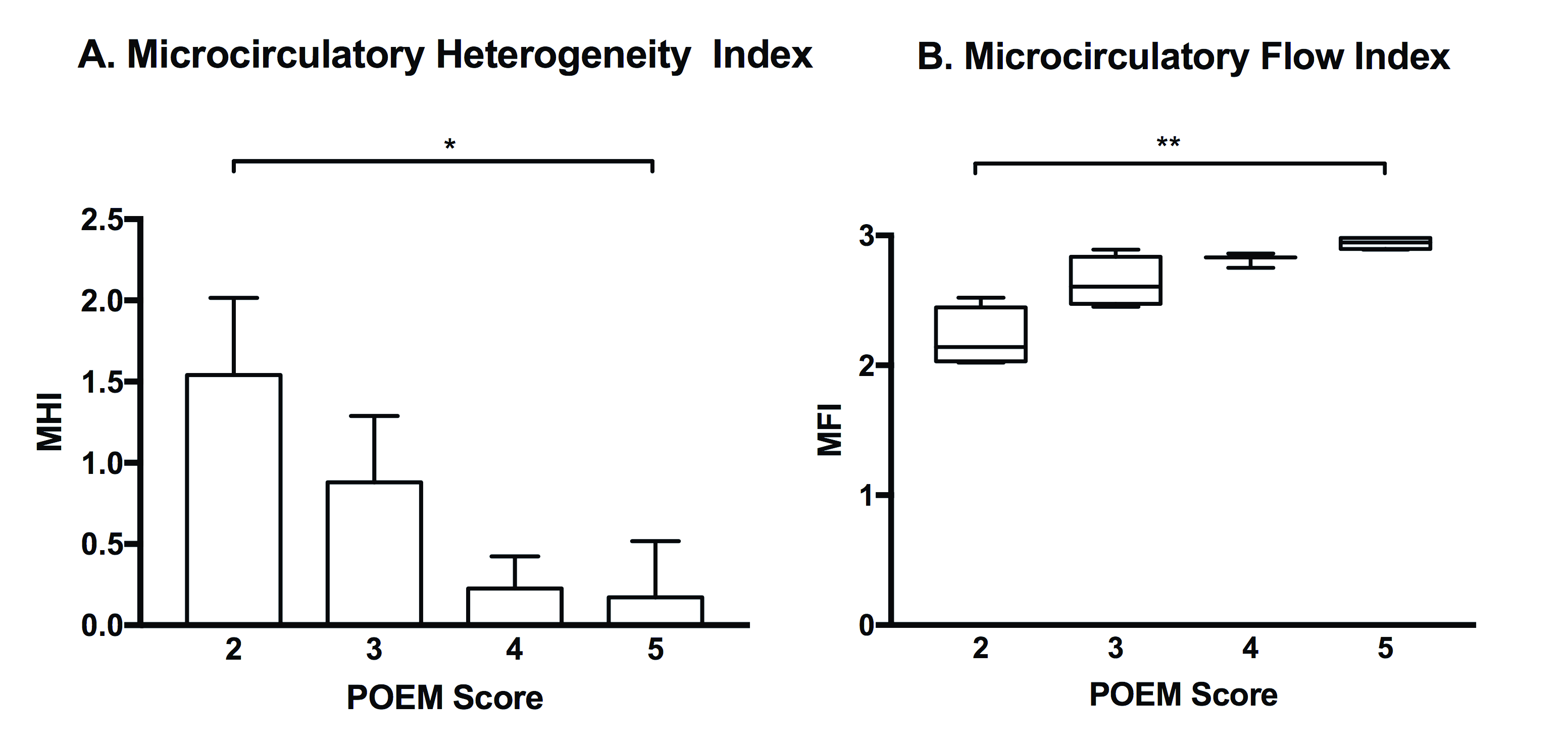

Supplement: Additional file 3: Figure S2. — Relationship between offline computer analysis of microcirculatory heterogeneity and flow indexes and POEM scores with the POEM score as a categorical variable (*p < 0.05; **p < 0.01). (TIFF 278 kb) [file 13054_2016_1492_MOESM3_ESM.tiff]
